# Increased Polyamines Alter Chromatin and Stabilize Autoantigens in Autoimmune Diseases

**DOI:** 10.3389/fimmu.2013.00091

**Published:** 2013-04-17

**Authors:** Wesley H. Brooks

**Affiliations:** ^1^Department of Chemistry, University of South FloridaTampa, FL, USA

**Keywords:** polyamines, NETosis, autoimmune disease, peptidylarginine deiminase, nuclear aggregates of polyamines, neutrophil extracellular TRAPs

## Abstract

Polyamines are small cations with unique combinations of charge and length that give them many putative interactions in cells. Polyamines are essential since they are involved in replication, transcription, translation, and stabilization of macro-molecular complexes. However, polyamine synthesis competes with cellular methylation for *S*-adenosylmethionine, the methyl donor. Also, polyamine degradation can generate reactive molecules like acrolein. Therefore, polyamine levels are tightly controlled. This control may be compromised in autoimmune diseases since elevated polyamine levels are seen in autoimmune diseases. Here a hypothesis is presented explaining how polyamines can stabilize autoantigens. In addition, the hypothesis explains how polyamines can inappropriately activate enzymes involved in NETosis, a process in which chromatin is modified and extruded from cells as extracellular traps that bind pathogens during an immune response. This polyamine-induced enzymatic activity can lead to an increase in NETosis resulting in release of autoantigenic material and tissue damage.

## Introduction

**Figure 1:**
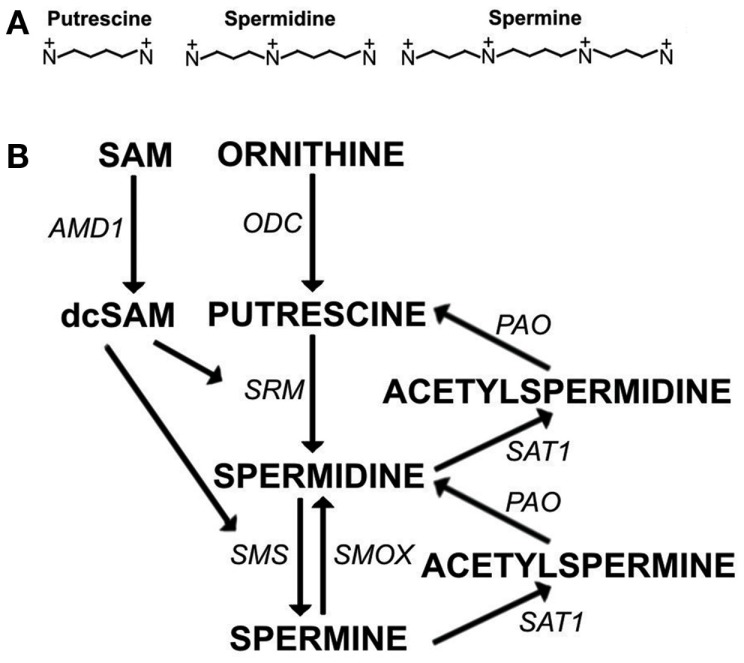
**The polyamine pathway**. **(A)** Polyamines at physiological pH: Putrescine (+2, ∼8Å), Spermidine (+3, ∼12Å), Spermine (+4, ∼16Å). **(B)** Polyamine synthesis and recycling. *S*-adenosylmethionine decarboxylase (AMD1) and ornithine decarboxylase (ODC) are rate limiting steps in polyamine synthesis. AMD1 decarboxylates *S*-adenosylmethionine (SAM) to decarboxylated SAM (dcSAM) so that dcSAM can provide aminopropyl groups added to putrescine to make spermidine by spermidine synthase (SRM) and added to spermidine to make spermine by spermine synthase (SMS). Spermine can be recycled to spermidine directly by spermine oxidase (SMOX). Spermine and spermidine can be recycled to spermidine and putrescine, respectively, by spermidine/spermine-*N*1-acetyltransferase (SAT1) followed by oxidation by polyamine oxidase (PAO).

Polyamines (putrescine, spermidine, and spermine) (Figure 1A) are essential for transcription, translation, replication, modulation of ion channels, modulation of receptor binding, and stabilization of many nucleoprotein complexes (Moinard et al., 2005; Pegg, 2009). The unique combination of length and high positive charge at physiological pH found in polyamines gives them the potential for many interactions with anions such as RNA, DNA, phospholipids, and ATP. However, excessive levels of polyamines have the potential to interfere with cellular processes and, therefore, polyamine synthesis must be controlled. For example, polyamine synthesis competes with essential cellular methylation for the methyl donor, *S*-adenosylmethionine (SAM). In eukaryotic cells, spermidine and spermine are usually present at 0.5–1.2 mM concentrations but putrescine is kept at only trace amounts when not needed since polyamine synthesis can be controlled by limiting putrescine. Putrescine is produced by a key enzyme in polyamine synthesis, ornithine decarboxylase (ODC) (Figure 1B). ODC is one of the most tightly controlled enzymes in cells with rapid turnover of ODC mRNA and protein and existence of an antizyme to help with ODC suppression (Pegg, 2006). Besides being the precursor for synthesis of higher polyamines, putrescine controls the self-processing conversion of SAM decarboxylase (AMD1) proenzyme to the active enzyme (Pegg, 2009). In addition, putrescine can bind an allosteric site in AMD1 that increases AMD1 activity eightfold converting SAM to decarboxylated SAM (dcSAM) (Bale et al., 2008). The dcSAM provides the amino propyl groups needed in polyamine synthesis. Cellular methyltransferases cannot use dcSAM, thus the need to keep putrescine and polyamine synthesis at low levels in order to control AMD1 activity and preserve SAM for methylation.

Increased levels of polyamines as well as increased polyamine synthesis and recycling have been seen in autoimmune diseases (Tetia et al., 2002; Karouzakis et al., 2012). The polyamines are usually bound non-covalently to major anions, such as DNA, RNA, and phospholipids, and only 2–15% of polyamines are free (Igarashi and Kashiwagi, 2010). Polyamine synthesis and recycling can greatly increase in response to cellular stress so that the free polyamines produced can bind and help stabilize disrupted macro-molecular complexes. Excessive cellular stresses could result in higher levels of polyamines. Another reason that cells need to keep polyamine levels under control is because polyamine degradation can generate toxic reactive molecules such as acrolein and hydrogen peroxide.

## Nuclear Aggregates of Polyamines

**Figure 2:**
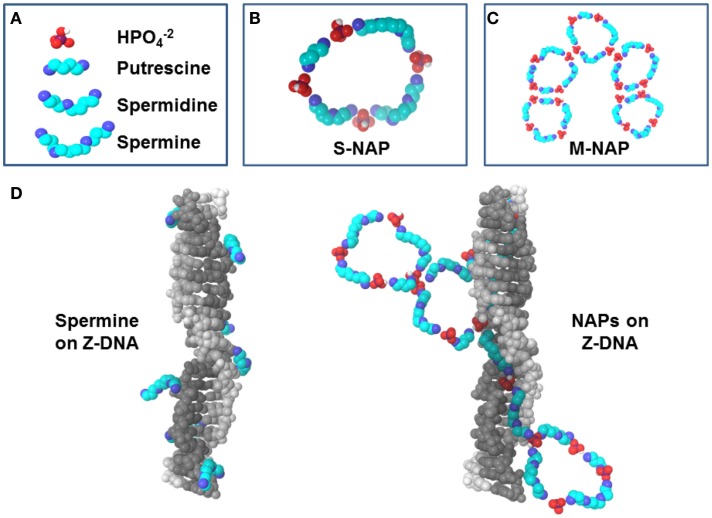
**Polyamines and NAPs**. **(A)** Components of NAPs. **(B)** Small nuclear aggregates of polyamines (s-NAP) proposed by D’Agostino et al. (2005) consists of phosphate ions with one putrescine, one spermidine, and two spermines. **(C)** Medium NAP (m-NAP), as proposed, consists of pentamers of s-NAPs. **(D)** Z-DNA stabilization by spermine versus NAPs. Left: Z-DNA can be co-crystallized with spermine [based on 2DCG.pdb (Wang et al., 1979) deposited in the Protein Data Bank, www.rcsb.org (Berman et al., 2000)]. Note how spermine molecules interact individually with DNA and could be displaced easily by nucleases. Right: proposed interaction of NAPs with Z-DNA. An s-NAP could bind in the Z-DNA minor groove, aligning other polyamines in the NAP to unroll into a growing stretch of Z-DNA. Polyamines from NAPs would reinforce each other in binding to DNA, making the DNA less vulnerable to nucleases.

Interactions of individual polyamines have typically been the focus of polyamine research but several key studies have begun the characterization of nuclear aggregates of polyamines (NAPs) (Pignata et al., 1999; D’Agostino and Di Luccia, 2002; D’Agostino et al., 2005). NAPs show a consistency as structures of approximately 1, 5, and 8 kDa which are identified as a small-size NAP (s-NAP), medium-size NAP (m-NAP), and large-size NAP (l-NAP), respectively. Similar structures are seen *in vivo* and *in vitro* (Iacomino et al., 2012). *In vitro* analysis has shown that polyamines slowly self-assemble with phosphate ions into the ring-like NAP structures (Figures 2A–C) (Iacomino et al., 2011). NAPs contain fixed ratios of polyamines and phosphate ions, believed to be primarily in the $HPO_{4}^{-2}$ state found in mildly basic physiological conditions. For the s-NAP (Figure 2B), the proposed structure is a single ring composed of one putrescine, one spermidine, and two spermine molecules, along with phosphate ions (D’Agostino et al., 2005). The proposed m-NAP structure (Figure 2C) is a pentamer of s-NAP rings. The proposed l-NAP structure (not shown) has multiple rings with a ratio of one putrescine, one spermidine, and one spermine along with phosphates ions.

Putrescine is key in initiation of NAP formation *in vivo* since its normally low levels would limit the rate of NAP formation. Also, in initiation of inter-monomer interactions (hydrogen bonding between phosphates) in going from s-NAP to m-NAP, the putrescine portion of the s-NAP would be important because any repulsion between the putrescine molecules is neutralized by the phosphates, whereas spermidine and spermine would still have internal amines with positive charges that could interfere with inter-monomer interactions. On the other hand, spermine in the NAP would be important since it would still have two internal positive charges available to initiate docking of the NAP to DNA or RNA (Iacomino et al., 2012). NAPs interacting with DNA, RNA, or other macro-molecular structures could stabilize autoantigenic conformations and protect the structures from nucleases and proteases allowing persistence of the autoantigens.

Nuclear aggregates of polyamines formed *in vitro* in the presence of DNA yield quite organized structures wrapped around the DNA, which could conceivably hamper nuclease digestion *in vivo* (Iacomino et al., 2011). However, when NAPs form with chromatin *in vivo*, the resulting complexes would be influenced by the presence of proteins, the differences in DNA content (AT-rich or GC-rich) and the presence of supercoiling stress. Polyamines could self-assemble into NAPs, bind DNA, and stabilize the normally transient left-hand coiling Z-DNA (Figure 2D) which is a form of negative supercoiling stress (i.e., unwinding of the right-hand coiling B-DNA form of the double helix) (Rich and Zhang, 2003). Particularly effective would be binding of spermine of an s-NAP in the narrow minor groove of Z-DNA, hydrogen bonding with the DNA phosphates on either side. Then, as negative supercoiling stress fluxes through the site and more local flipping to Z-DNA occurs transiently, the s-NAP will be perfectly aligned to unroll into the minor groove of the newly formed Z-DNA, stabilizing it as Z-DNA (D’Agostino et al., 2005). If that s-NAP is part of an m-NAP, then additional s-NAPs of the m-NAP could also be in alignment for rapid unfolding (Figure 2D). There could be a fast moving zipper effect of B-DNA to Z-DNA transition and stabilization by NAPs when there is a large flux of supercoiling stress released in chromatin. We should note that antibodies targeting Z-DNA forming sequences are found in systemic lupus erythematosus (SLE) and rheumatoid arthritis (RA) patients and Z-DNA forming elements are found in SLE sera (Sibley et al., 1984; Van Helden, 1985; Krishna et al., 1993).

**Figure 3:**
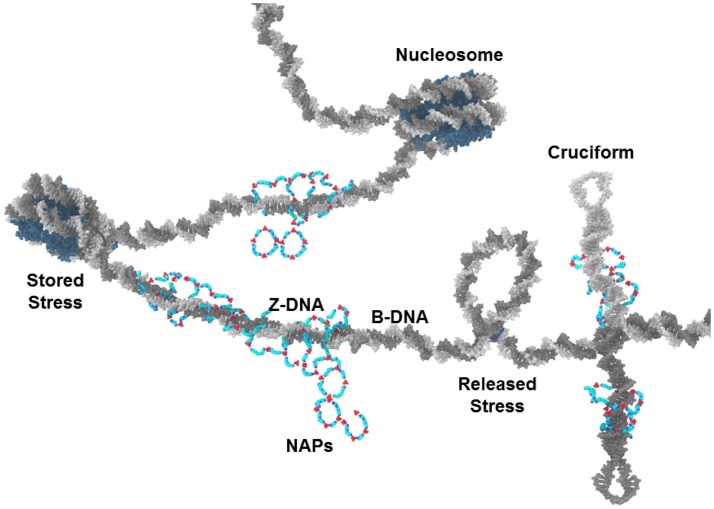
**NAPs and chromatin**. NAPs bound to chromatin capture negative supercoiling stress released from nucleosomes during NETosis. In this depiction, NAPs stabilize Z-DNA and cruciforms which are usually transient forms of negative supercoiling. These could become autoantigens when released as part of NETs.

Another form of negative supercoiling stress storage in DNA is the cruciform. Cruciforms can occur when the DNA double strands unwind, separate, and intra-strand homologous sequences hybridize (Figure 3) (Brázda et al., 2011). Alu elements, of which there are more than 10^6^ in the human genome and which have high G/C content, have the potential for intra-strand hybridization (as seen in the Alu domain of the 7SL RNA of the signal recognition particle) but almost always have a nucleosome positioned in the Alu DNA element that prevents the cruciform formation. Displacement or disruption of the nucleosome could allow cruciform formation that could be stabilized by NAPs. The NAPs could also provide protection from nucleases. We should note that, whereas Alu elements make up approximately 10% of the human genome, free DNA in sera of SLE patients is G/C rich and is 55% Alu DNA (Li and Steinman, 1989; Kreig, 1995).

## Limiting Factors: Negative Supercoiling Stress and Putrescine

The stabilization of Z-DNA and cruciforms by polyamines and NAPs in potentially nuclease resistant autoantigenic forms is limited by the availability of fluxing negative supercoiling stress and by the amount of putrescine that can initiate polyamine synthesis and NAP formation. Most DNA in chromatin is in B-DNA conformation, including the DNA in nucleosomes. Also, most DNA in chromatin is associated with nucleosomes. Z-DNA is less flexible than B-DNA and cannot bend sufficiently to be included in nucleosomes. In addition, cruciform formation requires strand unwinding, strand separation, and intra-strand hybridization, steps that are constrained by nucleosomes. Therefore, due to the abundance of nucleosomes, Z-DNA and cruciforms occur infrequently. Nucleosomes occur every 200 bp on average in humans with 145 bp wrapped around the histone octamer core and another approximately 55 bp in the linker DNA between nucleosomes. The DNA wrapped around the histone core is in B-DNA conformation but the DNA double strand forms a left-hand supercoil as it wraps around the histone core (Figure 3). This left-hand supercoil is, in effect, stored negative supercoiling stress. Each nucleosome contains approximately one negative supercoil. There are 3 × 10^9^ bp in the haploid human genome and, with a nucleosome on average every 200 bp, there are 30 × 10^6^ negative supercoils stored in the nuclear chromatin (diploid). Approximately 10^6^ of these negative supercoils are stored in positioned nucleosomes in Alu elements suppressing the cruciform formation potential of Alu elements. Major cellular events that impact chromatin, such as apoptosis, could rapidly release much of this negative supercoiling stress. As it fluxes through the chromatin, it could flip into Z-DNA, cruciforms, strand separation, reformation of nucleosomes, or be resolved by topoisomerases or protein binding. If the cellular event involves protein modifications or denaturation of histones and topoisomerases, then the balance can shift away from nucleosomes and topoisomerases toward the other forms of stress storage. Increased polyamines, especially in the form of NAPs, can help capture this shift in supercoiling stress.

The availability of NAPs depends on an increase in putrescine, as mentioned previously. Polyamines increase during S phase and in response to cell stresses, such as oxidative stress (Smirnova et al., 2012). There would be a temporary increase in putrescine before it is converted to spermidine and spermine.

Another way in which putrescine levels could become elevated in a cell is from a viral infection, such as an Epstein–Barr virus (EBV) infection. EBV is suspected of having a role in autoimmune diseases (Toussirot and Roudier, 2008). When EBV becomes active in a cell, it increases the activity of the c-Myc protein (Bajaj et al., 2008). The c-Myc protein modulates as much as 15% of gene expression throughout the genome. Among the genes with activity enhanced by c-Myc are genes involved in polyamine synthesis: ODC, spermidine synthase (SRM), and spermine synthase (SMS) (Bello-Fernandez et al., 1993; Nilsson et al., 2005; Hogarty et al., 2008). EBV-induced overexpression of ODC would lead to an increase in putrescine along with a subsequent decrease in SAM and cellular methylation since the putrescine would enhance AMD1 activity. Overall free polyamine availability would also increase with the enhanced expression of SRM and SMS. Other viruses may have actions and effects similar to EBV. Bacterial infections also could increase the available putrescine levels since bacteria freely produce putrescine without the constraints on ODC that eukaryotic cells have.

A third way in which putrescine levels could become elevated is increased activity and expression of spermidine/spermine-N1-acetyltransferase (SAT1), which is involved in recycling of polyamines. SAT1 is located at Xp22.1 on the X chromosome and is normally suppressed on the inactive X but expressed from the active X. SAT1 can undergo super induction (greater than 100-fold increased expression) in the presence of reactive oxygen species (ROS) (Chopra and Wallace, 1998). This can create an abundance of acetylated polyamines, some of which would be transported out of the cell or oxidized to the next lower polyamine: acetylspermine to spermidine or acetylspermidine to putrescine. In addition, loss of X chromosome inactivation with subsequent overexpression of SAT1 from both X chromosomes has been proposed as a mechanism in autoimmune diseases (Brooks, 2012). With age, through many cell cycles and stresses, some of the X-linked genes can become overexpressed through loss of the epigenetic control established by X inactivation, which is dependent on methylation. SAT1 overexpression could lead to: excess putrescine through polyamine recycling; decreased SAM availability; and an abundance of acrolein from polyamine degradation. Acrolein-conjugated proteins are a biomarker in Sjögren’s syndrome (SjS) (Higashi et al., 2009). Increased SAT1 and polyamine recycling have recently been reported in RA (Karouzakis et al., 2012).

## NETosis

Extracellular release of nuclear components appears to have a role in their development as autoantigens and NETosis provides such exposure of nuclear components relatively intact compared to apoptosis or necrosis (Su and Pisetsky, 2009). Therefore, the recently discovered process of NETosis has drawn significant interest as potentially being involved in autoimmune diseases (Brinkmann et al., 2004; Fuchs et al., 2007; Knight and Kaplan, 2012; Darrah and Andrade, 2013). NETosis is a major cellular event that can bring about the rapid release of the negative supercoiling stress stored in the nucleus. In NETosis, a neutrophil is stimulated to modify its chromatin, primarily by citrullination of histones (Wang et al., 2009). This loosens the nucleosome’s hold on DNA and thereby releases the stored negative supercoiling allowing unraveling and expansion of the chromatin. Once the chromatin has been modified, the cell extrudes the chromatin into the local extracellular environment. In the case of neutrophils, this modified chromatin is referred to as a “neutrophil extracellular trap” or NET which binds pathogenic material to make it easier for macrophages to clear debris at an infection site. This process in neutrophils is referred to as NETosis but other cells, such as mast cells, can also undergo the general process referred to as ETosis. However, NETosis is most intriguing in relation to autoimmune diseases since neutrophils (a.k.a. granulocytes) are the most abundant of immune cells and are the first to arrive at an infection site to counter pathogens (Summers et al., 2010). Neutrophils originate from the bone marrow and can move into most tissues but they have a very short half-life of only a few hours before they terminate by apoptosis, necrosis, or other means.

NETosis can be induced in neutrophils by a number of factors including lipopolysaccharides from pathogens, interleukin-8 (IL-8), phorbol myristate acetate (PMA), and complement factor C5α (after priming by interferons I and II) (Wartha et al., 2007). We should note that EBV induces expression of IL-8 in neutrophils (McColl et al., 1997). And so an EBV infection, probably with a heavy viral load in cells, could potentially cause excessive NETosis activity and extracellular release of modified chromatin. Once NETosis is initiated, cytoplasmic granules merge with the neutrophil’s nucleus, releasing enzymes into the nucleus to modify the chromatin in preparation for extracellular release of the chromatin (Parker and Winterbourn, 2013). In addition, generation of ROS by NADPH oxidase activity adds to the denaturation of chromatin. The ROS could potentially cause super induction of SAT1, as mentioned previously.

## Peptidyl Arginine Deiminases

**Figure 4:**
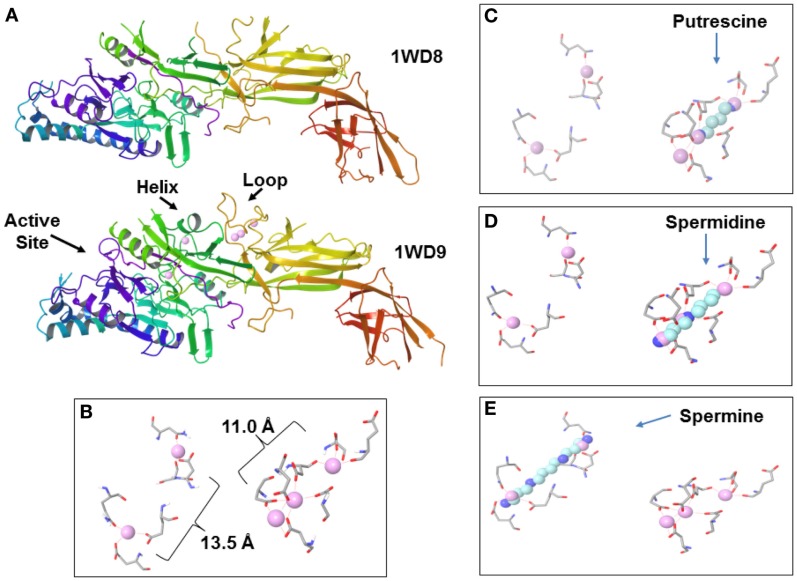
**Peptidylarginine deiminase 4 (PAD4) and polyamines**. **(A)** Inactive PAD4 without calcium (top, 1WD8.pdb; Arita et al., 2004) and active PAD4 (bottom, 1WD9.pdb; Arita et al., 2004) with bound calcium ions (pink spheres). Note the active site, stabilized helix and loop in active PAD4. **(B)** Calcium ions bound in PAD4 primarily by aspartic acid residues. Faint red dash lines indicate electrostatic interactions. **(C)** Putrescine superimposed over calcium ions. **(D)** Spermidine superimposed over calcium ions. **(E)** Spermine superimposed over calcium ions.

A key enzyme in the chromatin modification in NETosis is peptidylarginine deiminase 4 (PAD4, originally called peptidyl arginine deiminase V in humans) (Wang et al., 2009; Li et al., 2010). PAD4 localizes to euchromatin in the nucleus whereas other PADs are cytoplasmic (Nakashima et al., 2002). PAD4 is activated by binding of calcium ions (Figures 4A,B). Calcium is usually at very low levels in the cell (in the range of 100 nM to slightly greater than 1 μM) at which PAD4 is inactive whereas extracellular calcium is in the 1 mM range. In NETosis calcium stored in granules can be released into the nucleus to activate PAD4.

PAD4 converts the positively charged arginine residues in histones to neutral citrulline residues. This then loosens the hold of the histones on the DNA in nucleosomes, resulting in expansion of the modified chromatin due to self-repulsion of the negatively charged DNA and due to release of DNA supercoiling stress stored in the nucleosomes. The released stress can flux through the neighboring DNA facilitating unfolding of condensed higher-order chromatin structures, exposing more histones for modification. The modified chromatin is then extruded from the cell as a NET and spreads out to capture pathogens, such as bacteria and fungi (Wartha and Henriques-Normark, 2008). The neutrophil’s granules also release anti-microbial peptides into the nucleus, such as bactericidal permeability increasing (BPI) protein, and enzymes, such as elastase and myeloperoxidase (Wartha et al., 2007). These peptides and enzymes including PADs are released with the extracellular NET. The NET/pathogen complexes are then phagocytized by macrophages and the fragments are scrutinized by the adaptive immune system (T cells and B cells). Normally endogenous DNA and proteins should be tolerated and there should only be an adaptive immune reaction to exogenous pathogenic material. The presence of NAPs in the NETs could stabilize and protect autoantigens from degradation. Enzymes such as the PADs that are released during NETosis can act on the extracellular proteins encountered. For example, PADs released during NETosis could be responsible for the excessive amounts of citrullinated myelin basic protein seen in multiple sclerosis (MS) or the citrullinated collagen type II seen in RA.

The initiation of NETosis is believed to depend on an increase of calcium ions. However, since calcium levels in cells are usually low (sub μM) and the increase in nuclear levels of calcium appears to occur only in extraordinary situations, such as NETosis or apoptosis, it has been suggested that there may be other factors that can regulate PAD during routine activities, like gene control, that involves deimination (György et al., 2006). The published coordinates for PAD4 [1WD9.pdb (Arita et al., 2004) deposited in the Protein Data Bank, www.rcsb.org; Berman et al., 2000] display a very interesting possibility (Figure 4B). Five calcium ions bind PAD4, two near the active site and three toward the center of the enzyme. The distances approximate the lengths of the polyamines. It is proposed here that free polyamines could bind PAD4, and perhaps other PADs, and cause some level of citrullination activity in spite of low calcium levels (Figures 4C–E).

## The Hypothesis: “NAPs in NETs”

To summarize the hypothesis, it can be stated as: increased polyamines can cause potentially detrimental chromatin modifications and stabilize autoantigens. (1) Hypomethylation of chromatin can occur due to competition between polyamine synthesis and cellular methyltransferases for SAM. (2) Inappropriate induction of histone citrullination by PAD4, possibly activated by polyamines, can make neutrophils (and other cell types) more susceptible to initiation of NETosis (ETosis). (3) Alternate conformations of nucleic acids, nucleoproteins, and/or proteins can be stabilized in potentially autoantigenic forms when chromatin is disrupted, especially when polyamines and NAPs increase. When the modified chromatin is released from the cell, the alterations can persist through macrophage digestion and be interpreted by the immune system as autoantigens. In addition, PADs released with the modified chromatin can damage extracellular structures, such as the myelin sheath in MS and connective tissues in RA.

The possible activation of PADs by polyamines that would make neutrophils more sensitive to NETotic stimuli or more prone to initiate NETosis suggests a common mechanism for many of the autoimmune diseases. The autoantigens and tissue damage that arise and other disease-specific symptoms would result in part from the tissue location where the mechanism occurs. For example, in MS inflammation isolated behind the blood-brain barrier, tissue damage by released PADs is the main target whereas in SLE, a systemic disease with widespread circulation of antibody-autoantigen complexes, the nuclear autoantigens depositing in the kidneys and pericardium are usually a greater problem than localized tissue damage at other sites. The involvement of SjS secondary to SLE, MS, and RA and the fact that acrolein-conjugated proteins are a biomarker in SjS, may be attributable to polyamine involvement in these diseases.

The “NAPs in NETs” hypothesis suggests some interesting possibilities. For example, the female:male ratio of SLE patients is approximately 9:1 suggesting possible influence of the X chromosome and/or the X inactivation process. Alu elements have an important role in SLE according to the hypothesis. Whereas Alu elements account for approximately 10% of the human genome, they comprise only 8% of the X chromosome (Ross et al., 2005). However, the Alu composition jumps to ∼29% in the pseudo-autosomal region 1 (PAR1) of the X short arm and is ∼19% in the adjacent S5 region. Most Alu elements are silenced by a positioned nucleosome over the RNA polymerase III intragenic promoter boxes. These positioned nucleosomes would also suppress cruciform formation within the Alu DNA. This Alu DNA could form cruciforms when the nucleosomes are displaced during NETosis. With the extensive packaging in the inactive X chromosome which keeps greater than 75% of its genes silenced, there would be more stored supercoiling stress that could be rapidly released and stabilized in cruciforms or Z-DNA during NETosis, particularly in the PAR1 and S5 regions of the inactive X. The inactive X chromosome in females and Klinefelter’s males (XXY), but not in other males, could contribute to the female:male ratio in SLE.

## Future Perspectives

This “NAPs in NETs” hypothesis is compatible with several other hypotheses of autoimmune diseases. Proponents of those hypotheses may wish to consider the synergies. For example, the “hygiene hypothesis” attributes a protective role to helminth worms. The helminths take up polyamines and appear dependent on their host for polyamine synthesis (Sharma et al., 1991). This suggests helminths help modulate overall polyamine levels in the host. The “leaky gut” hypothesis fits well since bacteria in the intestines are a source of polyamines. A leaky gut could allow excessive amounts of polyamines to enter the circulation. The “molecular mimicry” hypothesis also fits since, for example, molecules mimicking IL-8 or PMA could trigger NETosis in neutrophils, especially if they have been potentiated by polyamine-activated PADs. “NAPs in NETs” also supports the “common cause” hypothesis of autoimmune diseases. Although “NAPs in NETs” places more emphasis on epigenetics, genetics-based hypotheses can align with “NAPs in NETs.” For example, some HLA types may be more permissive to EBV entry into cells, thereby creating lymphocytes with a heavier viral load and increase in viral induced polyamine synthesis (Li et al., 1997). One could envision EBV-infected cells at an infection site inducing IL-8 mediated polyamine-potentiated NETosis that releases more EBV, which infects more cells. A critical mass builds up to a persistent inflammation, the whole time PADs and autoantigens are being released extending the damage and response.

Although the “NAPs in NETs” hypothesis is complicated, it does suggest some obvious experiments. PAD activity could be analyzed in a range of concentrations of calcium and of polyamines. Polyamines alone may not activate PADs but they may greatly lower the threshold such that much lower concentrations of calcium can activate PADs. Another set of experiments could test the nuclease sensitivity of negatively supercoiled plasmid DNA with potential Z-DNA forming sequences (e.g., alternating purine-pyrimidine) in the presence/absence of NAPs. NAPs are interesting new phenomena to explore but increases in the individual polyamines also need further exploration. For example, immunization of rabbits with spermine alone induces autoantibodies targeting, DNA, and histones (Atanassov et al., 1993). Certainly drug discovery efforts to find new therapeutics to treat autoimmune diseases should intensify to target the PADs and enzymes involved in polyamine synthesis and recycling. The potential has been demonstrated by use of difluoromethylornithine (DFMO), an inhibitor of ODC, to suppress lupus-like symptoms in mouse models of lupus (Thomas and Messner, 1991).

## Conclusion

The “NAPs in NETs” hypothesis suggests that epigenetics and the innate immune response have significant roles in autoimmune diseases. This is quite different from the emphasis on genetics and possible abnormalities in the adaptive immune response that have been the focus of most autoimmune disease research over the past decades. Epigenetics and autoimmunity as a topic is receiving more attention recently as we try to understand the relationship between genetics and environmental factors in autoimmune diseases (Brooks et al., 2010).

What has been presented here as the “NAPs in NETs” hypothesis is actually a collection of hypotheses, each of which has its own merits, possible shortcomings, and open questions. Polyamine activation of PADs can be considered as one hypothesis. NAP stabilization of Z-DNA and/or cruciforms into autoantigens is another hypothesis. The importance of Alu elements, especially in PAR1 and S5 of the X chromosome, is yet another hypothesis. The discussion has focused mainly on neutrophils and NETosis but other cell types could be involved with similar problems from excess polyamines, loss of dosage compensation, inappropriate PAD activity, and/or ETosis (Goldman and Medina, 2013).

## Conflict of Interest Statement

The authors declare that the research was conducted in the absence of any commercial or financial relationships that could be construed as a potential conflict of interest.
